# Short Questionnaire for Workplace Analysis (KFZA): factorial validation in physicians and nurses working in hospital settings

**DOI:** 10.1186/s12995-017-0157-6

**Published:** 2017-05-12

**Authors:** Patricia Appel, Michael Schuler, Heiner Vogel, Amina Oezelsel, Hermann Faller

**Affiliations:** 1 Department of Medical Psychology and Psychotherapy, Medical Sociology and Rehabilitation Sciences, University of Würzburg, Klinikstrasse 3, 97070 Würzburg, Germany; 2 Center for Occupational Health and Psychology, hanza resources, Hammerbrookstr. 93, 20097 Hamburg, Germany

**Keywords:** KFZA, Mental health, Work-related stress, Hospital, Psychosocial workplace risk assessment, Validation

## Abstract

**Background:**

In recent years, there has been an increasing interest in psychosocial workplace risk assessments in Germany. One of the questionnaires commonly employed for this purpose is the Short Questionnaire for Workplace Analysis (KFZA). Originally, the KFZA was developed and validated for office workers. The aim of the present study was to examine the factorial validity of the KFZA when applied to hospital settings. Therefore, we examined the factorial structure of a questionnaire that contained all the original items plus an extension adding 11 questions specific to hospital workplaces and analyzed both, the original version and the extended version.

**Methods:**

We analyzed questionnaire data of a total of 1731 physicians and nurses obtained over a 10-year period. Listwise exclusion of data sets was applied to account for variations in questionnaire versions and yielded 1163 questionnaires (1095 for the extended version) remaining for factor analysis. To examine the factor structure, we conducted a principal component factor analysis. The number of factors was determined using the Kaiser criterion and scree-plot methods. Factor interpretation was based on orthogonal Varimax rotation as well as oblique rotation.

**Results:**

The Kaiser criterion revealed a 7-factor solution for the 26 items of the KFZA, accounting for 62.0% of variance. The seven factors were named: “Social Relationships”, “Job Control”, “Opportunities for Participation and Professional Development”, “Quantitative Work Demands”, “Workplace Environment”, “Variability” and “Qualitative Work Demands”. The factor analysis of the 37 items of the extended version yielded a 9-factor solution. The two additional factors were named “Consequences of Strain” and “Emotional Demands”. Cronbach’s α ranged from 0.63 to 0.87 for these scales.

**Conclusions:**

Overall, the KFZA turned out to be applicable to hospital workers, and its content-related structure was replicated well with some limitations. However, instead of the 11 factors originally proposed for office workers, a 7-factor solution appeared to be more suitable when employed in hospitals. In particular, the items of the KFZA factor “Completeness of Task” might need adaptation for the use in hospitals. Our study contributes to the assessment of the validity of this popular instrument and should stimulate further psychometric testing.

**Electronic supplementary material:**

The online version of this article (doi:10.1186/s12995-017-0157-6) contains supplementary material, which is available to authorized users.

## Background

Analyses and monitoring of risks and hazards at the workplace are important tools to achieve and maintain healthy working conditions. In recent years, there has been a growing awareness of mental health aspects, which in Germany is also reflected by an amendment to the Safety and Health at Work Act (cf. section 5 of the Occupational Health and Safety Act, number 6). This has led to an increasing interest in Psychosocial Workplace Risk Assessments i.e., a systematic evaluation of health risks caused by workplace conditions. However, they are not often put into practice [1] resulting in a significant lack of experience [2]. Therefore, the practical aspects as the selection of suitable tools that capture all relevant psychosocial factors as defined below [3] and their implementation are gaining importance.

### Tools for psychosocial workplace risk assessments

Psychosocial factors in the workplace, such as work intensity, job control, emotional demands or social support, may influence mental health and have an impact on staff turnover and sickness absence rate. A guideline that describes all the relevant factors and acts as a compass for the relevant authorities has been published by the Joint German Occupational Safety and Health Strategy (GDA) [3]. As a first step, it is often recommended to use standardized written staff surveys to obtain a rough analysis. It has proven practical to employ an instrument with a maximum number of 40 questions, which take no longer than 20 min to be completed [4].

One of the questionnaires that fulfil these recommendations is the Short Questionnaire for Workplace Analysis (KFZA) developed by Prümper et al. [5]. It is listed in the toolbox of the Federal Institute of Work Safety and Occupational Medicine (BAuA). Originally, the KFZA was developed and validated for the analysis of working conditions at office workplaces, but it has been described as a universal tool applicable to all types of workplaces, industries and occupations [6, 7]. Both, the KFZA as such and modified versions that were integrated in a larger set of assessments, have been used in several previous studies in hospital workplaces [8–10].

### Working conditions in hospitals

About 10% of the workforce in Germany are employed in the health care system [11]. Analyses by health care insurances show that health care professionals tend to have the highest rates of health-related incapacity for work [12]. Multiple studies have shown that stress-related working conditions, such as time pressure, workload, interruptions of workflow, difficulties with patients or conflicts with co-workers and supervisors, are common in hospital workplaces [13–15] and may lead to reduced psychological and physical well-being [16, 17]. In addition, it is worth recalling that these working conditions can have negative consequences for patient outcome [16, 18].

As hospital workplaces are often characterized by stress-related working conditions and an increasingly challenging financial situation [19], it is especially important to choose a survey that is short, economic and practical. We therefore aimed to examine the suitability of the KFZA for this purpose by analyzing the factorial validity when applied to hospital settings. In the present study we used an extended version of the questionnaire with additional questions specific to hospital workplaces. The factorial structure of both the original and the extended versions were examined.

## Methods

The legally mandated Psychosocial Workplace Risk Assessments were conducted by a center for occupational medicine over a 10-year period between 2004 and 2014 and yielded questionnaire data of a total of 1731 hospital employees. As anonymous data collected as part of routine practice were analyzed, ethical approvement was not deemed necessary by the local ethics committee.

### Sample and procedure

The participating hospitals were located in northern Germany and the employees worked at regular wards, intensive care, intermediate care, emergency rooms and surgical departments.

Physicians and nurses from different disciplines were surveyed representing surgery, internal medicine, pediatrics, gynecology, urology, anesthesiology, neurology, orthopedics, ophthalmology, ENT, psychiatry and palliative care.

In preparation of the survey the employees were informed about purpose and procedure of the Psychosocial Workplace Risk Assessment and were given the opportunity to ask questions. These informative meetings were held by occupational psychologists and occupational physicians and were complemented by information letters. Participation was voluntary.

Please note, given that the data was collected in small group samples, demographic data was often not collected to protect the anonymity of the respondents due to requests by the work council or by data protection officers.

### Questionnaire

The self-administered questionnaire applied was based on the Short Questionnaire for Workplace analysis (KFZA), which consists of 26 items and 11 scales [5], and was extended adding 11 items that covered aspects lacking in the original KFZA and that are either of general importance or specific to hospital workplaces. The 11 scales of the KFZA are assigned to four aspects of work: “Job Content”, “Resources”, “Stressors” and “Organizational Culture”. An additional table shows work aspects, KFZA scales and items including additional items (see Additional file 1: Table S1).

The scales that focus on “Job Content” are *Variability* and *Completeness of Task*. The scale *Variability* contains three items (VS1-VS3), one example is “I can fully apply my knowledge, skills and abilities at work”. *Completeness of Task* is covered by two items (GH1/GH2), for example “My work allows me to complete products from beginning to end”.

Three of the scales cover “Resources”: *Job Control*, *Social Support* and *Cooperation*, each embracing three items. One example for the items of *Job Control* (HS1-HS3) is “I can determine the sequence of my job tasks”, for *Social Support* (SR1-SR3) “In difficult work situations I can rely on my co-workers” and for *Cooperation* (ZU1-ZU3) “My work requires close cooperation with other people in the department”, respectively.

Four scales focus on “Stressors”, including two items each. One example for *Qualitative Work Demands* (QL1, QL2) is “The demands that are made on my concentration are usually too high”, for *Quantitative Work Demands* (QN1, QN2) “I frequently work under time pressure”, for *Work Disruptions* (AU1, AU2) “I am repeatedly interrupted during my actual work” and for *Workplace Environment* (UB1, UB2) “There are unfavorable environmental conditions at my workplace, such as noise, air, dust”, respectively.

The remaining two scales embracing two items each cover the aspect “Organizational Culture”. One of the items of *Information and Participation* (IM1, IM2) is “We are sufficiently informed about important things and events that concern our department”, one example for *Benefits* (BL1, BL2) is “Our department provides good training opportunities”.

For the extended version the items of the KFZA were complemented by 11 items which add one item to the domain *Cooperation* (ZU4, “The various professional groups cooperate well”) and address the 3 additional domains *Work Equipment* (AM, “I have sufficient and adequate work equipment”), *Emotional Demands* (EB1-EB3, for example “My work is emotionally taxing”) and *Consequences of Strain* (FB1-FB6, for example “Due to work-related issues, I experience disordered sleep”), i.e. effects of psychosocial factors.

Each item was rated on a 5-point Likert scale.

### Statistical analysis

All analyses were performed with SPSS Statistics 23 and MS Office Excel 2013.

Means and standard deviations of the items were calculated. Regarding missing data, the effect of the variation in questionnaire versions was reported. Data sets with missing data were excluded listwise. We tested the effect of exclusion by comparing the group of analyzed data sets to the group of deleted data sets concerning the distribution of profession, gender, age and duration of department affiliation.

Due to the fact that the items ZU1, ZU3 and QL1 were only presented to a part of the sample, the responses to these questions were missing remarkably often. In some questionnaire versions item ZU1 (“My work requires close cooperation with other people in the department”) was deliberately left out because these circumstances apply to the majority of workplaces in a hospital. Item ZU3 (“I always receive feedback *by my supervisors and co-workers* about the quality of my work”) and item QL1 (“In my work, there are things that are too complicated”) were sometimes replaced by variations which divided these questions into two sub-questions: ZU3a (“I always receive feedback *by my supervisors* …”), ZU3b (“I always receive feedback *by my co-workers* …”), QL1a (“In my work, there are things that are too complicated due to *ambiguous process procedures*”) and QL1b (“… due to *insufficient training*”).

Thus, the accumulation of missing data was caused by variations in questionnaire versions instead of refusal to reply.

To examine the factor structure of the 26 items (37 items in the extended version), we conducted an exploratory factor analysis through principal component factoring method. The number of factors was determined using both the Kaiser criterion and scree-plot methods. Factor interpretation was based on the orthogonal Varimax rotation method as well as a direct oblimin method with an oblique rotation. Correlation coefficients among the extracted factors based on oblique rotation were calculated. The internal consistency of the scales was analyzed through Cronbach’s α coefficient of reliability.

## Results

### Sample characteristics

The data set of 1731 hospital employees included 476 physicians and 1255 nurses. A listwise exclusion of data sets with missing data left the data of 1163 questionnaires (1095 for the extended version) remaining for factor analysis. The following sociodemographic characteristics are listed in valid percent: 76.5% of the respondents were female, 23.5% male (*n* = 490 missing values). Regarding the age of the respondents 16.3% were 20-29 years old, 31.9% 20-39, 31.3% 40-49 and 20.4% 50-65 years old (*n* = 347 missing values). The duration of department affiliation amounted to 1-5 years in 39.6%, 5-10 years in 24.6% and >10 years in 35.8% (*n* = 392 missing values).

Comparing the analyzed sample to the deleted one, we found that the analyzed sample contained significantly more nurses, male and younger participants as well as more participants with a department affiliation of 1-5 years or more than 10 years.

### Factor analysis of the KFZA

The value of the Kaiser-Meyer-Olkin measure of sampling adequacy (0.87) and a significant Bartlett’s test of sphericity (*p* < 0.001, df = 325, chi-square = 10,603.331) indicated a high factoriability for the sample.

Principal component factor analysis was performed. The Kaiser criterion revealed a 7-factor solution for the 26 items of the KFZA, accounting for 62.0% of variance, whereas Cattell’s scree-plot indicated a 4-factor solution accounting for 49.0% of variance. As extracting too few factors is considered to be a more serious error than extracting too many [20], we opted for the 7-factor solution.

First, we applied Varimax orthogonal rotation enabling us to compare our results with the empirical scales obtained by Prümper et al. [5], which were based on a two-step factor analytical procedure. Table 1 shows the rotated matrix of components. As all factor loadings for item GH2 were below 0.40, it was assigned to factor II on which it showed the highest loading (0.36). An additional oblique rotation yielded a comparable factorial structure (data not shown).

**Table 1:** Factor loadings (orthogonal solution)

KFZA items	Component (% of variance)						
	I (24.6)	II (10.1)	III (7.4)	IV (6.9)	V (4.6)	VI (4.4)	VII (4.0)
SR1 Social support by co-workers	*0.77*	0.05	0.11	−0.07	0.11	0.06	0.22
SR3 Social cohesion within the department	*0.73*	0.11	0.31	−0.01	0.09	0.09	0.06
SR2 Social support by supervisors	*0.70*	0.03	0.38	−0.03	0.10	0.06	0.13
ZU2 Opportunity for social exchange with co-workers	*0.63*	0.24	0.03	0.29	−0.05	0.03	−0.11
ZU3 Feedback from supervisors and co-workers	*0.61*	0.17	0.24	0.24	−0.04	0.09	−0.17
ZU1 Necessity of cooperation	*0.53*	−0.05	−0.07	−0.30	0.04	0.31	0.23
HS1 Influence on sequence of activities	0.10	*0.86*	0.13	0.01	0.00	0.07	0.12
HS3 Influence on work load and procedures	0.10	*0.85*	0.19	0.03	0.05	0.11	0.08
HS2 Influence on work content	0.09	*0.84*	0.14	0.03	0.05	0.16	0.05
GH2 Completeness of product	0.33	0.36	0.08	0.20	0.20	0.31	−0.04
IM2 Consideration of employee input	0.22	0.12	*0.74*	0.05	0.04	0.12	0.00
IM1 Information about organizational developments	0.26	0.14	*0.73*	0.00	0.17	0.01	−0.03
BL1 Continuous education	0.08	0.10	*0.71*	0.03	0.11	0.24	0.08
BL2 Opportunities for advancement	0.11	0.12	*0.64*	0.09	−0.02	0.16	−0.09
QN2 Workload	0.04	0.06	0.05	*0.82*	0.14	0.08	0.19
QN1 Time pressure	0.10	0.09	0.01	*0.79*	0.14	0.07	0.22
AU2 Interruptions of workflow	−0.03	−0.06	0.11	*0.62*	0.41	0.07	−0.17
UB2 Insufficient work space and equipment	0.08	0.08	0.03	0.07	*0.83*	0.01	0.09
UB1 Unfavourable physicochemical conditions	0.11	0.06	0.07	0.20	*0.76*	0.06	0.04
AU1 Lack of information, work materials or equipment	0.00	0.01	0.20	0.34	*0.47*	−0.05	0.13
VS3 Variety of tasks	−0.03	0.01	0.13	−0.03	−0.05	*0.78*	−0.01
VS2 Use of knowledge, skills and ability	0.20	0.24	0.27	0.12	0.08	*0.58*	0.07
VS1 Learning new skills	0.16	0.18	0.39	0.17	−0.04	*0.56*	−0.06
GH1 Visibility of task accomplishment	0.25	0.35	0.07	0.07	0.13	*0.48*	0.03
QL1 Excessive complexity of tasks	0.06	0.14	−0.04	0.03	0.08	−0.01	*0.83*
QL2 Excessive demands on concentration	0.12	0.08	0.00	0.35	0.10	0.03	*0.74*

*Abbreviations: KFZA* Kurzfragebogen zur Arbeitsanalyse (Short Questionnaire for Workplace Analysis)

*﻿Note*: Factor loadings >0.4 are presented in italics

Substantive considerations supported the number of seven factors: The first factor (24.6% of total variance) contained the complete set of items from the KFZA factors *Social Support* (SR1-SR3) and *Cooperation* (ZU1-ZU3) and thus was labeled ***Social Relationships*** *.* The second factor (10.1%) included all items from the KFZA factor *Job Control* (HS1-HS3) and one additional item from *Completeness of Task* (GH2) “My work allows me to complete products from the beginning to the end”. Item GH2 is not precisely fitting to a hospital workplace and loaded on various factors with loadings lower than 0.40. This question seems to cover the ability to perform nursing measures or medical interventions from the beginning to the end. Therefore, item GH2 can be assigned to aspects that concern control over demands and tasks at work and the second factor was named ***Job Control*** *.* Factor III (7.40%) contained all items from the KFZA factors *Information and Participation* (IM1, IM2) and *Benefits* (BL1, BL2) and thus was labeled ***Opportunities for Participation and Professional Development***. The fourth factor (6.91%) included all items from the KFZA factor *Quantitative Work Demands* (QN1, QN2) and one additional item from *Work Disruptions* (AU2). As the number of interruptions can be subsumed under the aspect of quantitative work demands, we kept the label ***Quantitative Work Demands***. Factor V (4.64%) included all items from the KFZA factor *Workplace Environment* (UB1, UB2) and one item from *Work Disruptions* (AU1) “I often do not have the required information, materials, or tools at my disposal”. Lack of information and equipment can also be assigned to aspects of workplace environment which is why the factor was labeled ***Workplace Environment***. The sixth factor (4.38%) revealed a picture somewhat similar to that of the second factor. It contained all items from the KFZA factor *Variability* (VS1-VS3) and the other item from *Completeness of Task* (GH1). Again, item GH1 is not precisely fitting a hospital workplace. On account of the predominant variability component of most items of this factor, the label ***Variability*** was maintained. Lastly, factor VII (4.00%) included both items from the KFZA factor *Qualitative Work Demands* (QL1, QL2). Therefore, the label ***Qualitative Work Demands*** was retained. In summary, the seven determined factors correspond quite well with the original KFZA factors.

Table 2 shows correlations among the seven extracted factors based on the additional oblique rotation. Correlation coefficients tend to be low and range from *r* = |0.04| (Factor III vs. Factor VII) to r = |0.29| (Factor IV vs. Factor V).

**Table 2:** Correlations among extracted factors (oblique solution)

	SR	JC	OPPD	QNWD	WE	VAR	QLWD
Social Relationships (SR)	--						
Job Control (JC)	0.26	--					
Opportunities for Participation and Professional Development (OPPD)	−0.28	−0.26	--				
Quantitative Work Demands (QNWD)	−0.05	−0.16	0.14	--			
Workplace Environment (WE)	−0.15	−0.12	0.19	0.29	--		
Variability (VAR)	−0.26	−0.28	0.27	0.05	0.08	--	
Qualitative Work Demands (QLWD)	−0.14	−0.07	−0.04	0.01	0.16	0.04	--

### Reliability

We estimated the reliability of the scales on the basis of Cronbach’s α. The scales had 2 to 6 items each and Cronbach’s α reliability coefficient ranged from 0.63 to 0.80 (Table 3). The internal consistency of the scales *Social Relationships, Job Control*, *Opportunities for Participation and Professional Development* and *Quantitative Work Demands* can thus be regarded as satisfying. For the scales *Workplace Environment*, *Variability* and *Qualitative Work Demands* values of Cronbach’s α ranged only from 0.63 to 0.70. However, application is still acceptable for the use in larger samples to investigate scientific questions [21].

**Table 3:** Descriptive statistics and internal consistency of the scales of the KFZA (orthogonal solution)

Scales	Eigenvalues	M	SD	Cronbach’s α	Number of items
Social Relationships	3.10	3.53	0.46	0.80	6
Job Control	2.72	3.17	0.24	0.80	4
Opportunities for Participation and Professional Development	2.69	2.84	0.57	0.76	4
Quantitative Work Demands	2.28	2.34	0.19	0.76	3
Workplace Environment	1.87	2.92	0.29	0.65	3
Variability	1.87	3.63	0.22	0.68	4
Qualitative Work Demands	1.59	3.62	0.17	0.63	2

*Abbreviations: KFZA* Kurzfragebogen zur Arbeitsanalyse (Short Questionnaire for Workplace Analysis)

### Factor analysis of the extended version

In a second step, we conducted a factor analysis as described above of the 37 items of the extended version including 11 additional items specific to hospital workplaces. Again, the value of the Kaiser–Meyer–Olkin measure of sampling adequacy (0.88) and a significant Bartlett’s test of sphericity (*p* < 0.001, df = 666, chi-square = 14,472.214) indicated a high factoriability.

The Kaiser criterion yielded a 9-factor solution accounting for 59.5% of the variance. Using Varimax orthogonal rotation, all factor loadings were above 0.40.

As Factor I included the one additional item of the original domain *Cooperation* (ZU4) and the six items (ZU1-ZU3, SR1-SR3) of the previously determined factor ***Social Relationships,*** this label was maintained. The second factor contained the additional items from the domain *Consequences of Strain* (FB1-FB6) and was therefore named ***Consequences of Strain***. Factor III – contrary to the previously determined factor II – included only the complete set of items from the KFZA factor *Job Control* (HS1-HS3) and was labeled ***Job Control.*** Factor IV (IM1, IM2, BL1, BL2) and factor V (QN1, QN2, AU2), respectively, match the factors identified before, ***Opportunities for Participation and Professional Development*** and ***Quantitative Work Demands***. The sixth factor contained the additional item of the domain *Work Equipment* (AM) and the three items (UB1, UB2, AU1) of the previously determined factor ***Workplace Environment***. As the availability of adequate working equipment is also part of the environment of a workplace, this name was kept. Factor VII included the additional items from the domain *Emotional Demands* (EB1-EB3) and item GH2 “My work allows me to complete products from the beginning to the end” which before was assigned to the second factor *Job Control.* As this item seems to cover the aspect that an employee has the possibility to accompany patients from the beginning to the end of their stay and witness their recovery it might operate as relief of strain. Therefore, factor VII was labeled ***Emotional Demands*** *.* Factor VIII (VS1-VS3, GH1) and Factor IX (QL1, QL2), respectively, match the previously determined factors ***Variability*** and ***Qualitative Work Demands*** *.* Thus, the nine factors of the extended version correspond well with the seven factors of the original KFZA version that emerged from our study.

### Reliability of the extended version

Again, we estimated the reliability of the nine scales on the basis of Cronbach’s α which ranged from 0.60 to 0.87 (Additional file 2: Table S2). The internal consistency of three scales (*Social Relationships, Consequences of Strain, Job Control)* can be regarded as good and of two scales (*Opportunities for Participation and Professional Development, Quantitative Work Demands)* as satisfying, respectively, while for four scales (*Workplace Environment*, *Emotional Demands*, *Variability*, *Qualitative Work Demands)* values of Cronbach’s α ranged from 0.60 to 0.70.

## Discussion

Our study examined the factorial validity of the KFZA, a questionnaire developed and validated for office workers [5], plus an extended version of this questionnaire, in hospital settings. Our results suggest a 7-factor solution instead of the 11 factors originally proposed by the authors of the KFZA. A comparison can be seen in Table 4.

**Table 4:** Comparison of original KFZA factors and factors determined in this study

Work aspects	KFZA factor	Items (item code)	Factors determined in this study
Job Content	Variability	Learning new skills (VS1)	VI Variability
		Use of knowledge, skills and ability (VS2)	
		Variety of tasks (VS3)	
	Completeness of Task	Visibility of task accomplishment (GH1)	
		Completeness of product (GH2)	II Job Control
Resources	Job Control	Influence on sequence of activities (HS1)	
		Influence on work content (HS2)	
		Influence on work load and procedures (HS3)	
	Social Support	Social support by co-workers (SR1)	I Social Relationships
		Social support by supervisors (SR2)	
		Social cohesion within the department (SR3)	
	Cooperation	Necessity of cooperation (ZU1)	
		Opportunity for social exchange with co-workers (ZU2)	
		Feedback from supervisors and co-workers (ZU3)	
Stressors	Qualitative Work Demands	Excessive complexity of tasks (QL1)	VII Qualitative Work Demands
		Excessive demands on concentration (QL2)	
	Quantitative Work Demands	Workload (QN2)	IV Quantitative Work Demands
		Time pressure (QN1)	
	Work Disruptions	Interruptions of workflow (AU2)	
		Lack of information, work materials or equipment (AU1)	V Workplace Environment
	Workplace Environment	Unfavourable physicochemical conditions (UB1)	
		Insufficient work space and equipment (UB2)	
Organizational Culture	Information and Participation	Information about organizational developments (IM1)	III Opportunities for Participation and Professional Development
		Consideration of employee input (IM2)	
	Benefits	Continuous education (BL1)	
		Opportunities for advancement (BL2)	

*Abbreviations: KFZA* Kurzfragebogen zur Arbeitsanalyse (Short Questionnaire for Workplace Analysis)

The comparison of these two sets of factors shows that only the factor ***Qualitative Work Demands*** turned out to be an exact match. In addition to this factor, we found six factors instead of 10. The items of the original distinct factors *Social Support* and *Cooperation* combined to form a factor that was labeled ***Social Relationships*** *.* However, we acknowledge that this is based on our strictly empirical approach. Another approach might have been to retain the original factor structure based on theoretical constructs, that is, preserving the subscales found by Prümper et al. [5] instead of merging them, as the loadings of items from two specific scales in the original paper on one factor in our analysis may well be the result of these two scales being related in hospital settings. Likewise, the items of *Information and Participation* and *Benefits* built a factor which was named ***Opportunities for Participation and Professional Development***. In this case, our results align with the empirical structure obtained by Prümper et al. [5].

The items of the original factors *Completeness of Task* and *Work Disruptions* did not load on independent factors but divided up to load on four other factors. One item of *Completeness of Task* was assigned to the factor ***Variability***, the other item to ***Job Control.*** The low factor loadings of 0.48 and 0.36, respectively, suggest that the wording of these items is not optimally suitable for the use in hospitals and they might have been interpreted in different ways. An example covered by item GH1 (“From the outcomes of my work I can tell whether I did a good job or not”) might be that despite a correctly performed chemotherapy a patient may develop metastases. Likewise, item GH2 can be interpreted differently and covers various aspects as described above in the results section. One of the two items of *Work Disruptions* (AU2) was assigned to ***Quantitative Work Demands***, the other one (AU1) to ***Workplace Environment***. Given that quantitative demands are the biggest contributing factor to frequent work disruptions (e.g., patients calling, phones ringing) this factor solution has face validity. Therefore, item AU2 “Interruptions of workflow”, with a factor loading of 0.62, seems to be a useful complement to the items of the factor ***Quantitative Work Demands***. In contrast, item AU1 (“Lack of information, work materials or equipment”) describes a form of work disruptions less commonly found in hospitals and loads merely at 0.47 on the factor ***Workplace Environment***. A reason for this might be this item being similar to the item “Insufficient work space and equipment”.

For the seven scales we obtained, Cronbach’s α reliability coefficient ranged from 0.63 to 0.80, whereas values for the 11 scales in the original publication ranged from 0.40 to 0.76. When calculating Cronbach’s α, it is important to keep in mind that it increases with the number of items, such that in our analysis the number of items per scale was 2-6, while in the original analysis with 11 scales this value was 2-3. However, Cronbach’s α showed also an acceptable value for the scale ***Qualitative Work Demands,*** which consisted of the identical items as the original factor.

Most correlation coefficients among the seven factors turned out to be low, i.e. r < |0.20|. This is compatible with the high correspondence between the solutions based on Varimax rotation and on oblique rotation, respectively, with the former assuming statistical independence between the factors. Indeed, some of these correlations appear plausible, e.g., the highest positive correlation (0.29) for the scales ***Quantitative Work Demands*** and ***Workplace Environment,*** as environmental circumstances such as insufficient work space and equipment may lead to higher work demands like interruptions of workflow and time pressure. One of the highest negative correlations (−0.28) was found between the scales ***Variability*** and ***Job Control***. This might be due to the fact that a workplace that is characterized by a diverse and varied task profile may at the same time be quite unpredictable and difficult to plan. However, it should be noted that if scales correlate due to common causes, reaching scales with low coefficients may not be a goal in itself.

The factor analysis of the extended version of the questionnaire yielded a 9-factor solution (Additional file 3: Table S3). Of the seven previously determined factors, four were identified as consistent: ***Qualitative Work Demands***, ***Quantitative Work Demands***, ***Opportunities for Participation and Professional Development*** and ***Variability***. Some of the additional items produced two new factors: ***Consequences of Strain***, consisting of the six supplementary items from this domain, and ***Emotional Demands*** including the corresponding supplementary items and item GH2 of *Completeness of Task* that had been assigned to ***Job Control*** before. Again, this item showed a low factor loading of 0.46. Thus, the factor ***Job Control*** was reduced to the original three items of the KFZA. The factors ***Social Relationships*** and ***Workplace Environment*** were enhanced fittingly by one additional item each from the domains *Cooperation* respectively *Work Equipment.* Taken together, items that loaded on the same factor in our analysis of the KFZA remained assigned to this factor in the analysis of the extended version, except for item GH2. With a number of 2 to 7 items per scale, Cronbach’s α ranged from 0.63 to 0.87, with the scale ***Job Control*** showing highest internal consistency.

In summary, we were able to replicate the overall content-related structure found by the authors of the KFZA quite well, despite the reduced number of factors. The differences in the factorial structure can be explained by the differences in the specific stress-related working conditions, as detailed below.

The working conditions of hospital staff are characterized by a general increase in workload, by shift work, time pressure and frequent overtime, a high degree of responsibility, work disruptions, only partially predictable amount of work, hierarchical structures with deficits in communication and impairment through increasing economic interests [22–24]. In contrast, the factor analysis conducted by the authors of the KFZA was based on a sample of office workers. Whereas the main focus of hospital staff is working with human beings and mistakes and failures can lead to vital consequences, the work in an office is focused on administrative and organizational tasks and is characterized by the overall use of a computer [25]. Office workers report to experience especially time pressure, work disruptions and, besides a general increase in workload, primarily an increase in the flood of information by email [26].

However, in spite of these differences, there are also a number of similarities between the two fields that might contribute to the similarities of the results. For example, in both fields female employees predominate, with a proportion of two thirds in offices and 85% among nursing staff [27, 28]. Even among hospital physicians, the percentage of female doctors is growing and amounted to 46 – 56% in 2013 [29]. Further similarities are – as mentioned above – the dominance of time pressure, work disruptions and a general increase in workload, which correspond to universal changes in working environment [30].

Recently, Keller et al. [31] have presented results from a factorial analysis of a questionnaire specifically designed for hospital settings, the “Instrument for stress-related job analysis for hospital physicians” (ISAK-K). The size of this questionnaire containing 30 items is similar to that of the KFZA. Comparison of our determined KFZA factors with the 14 factors of the ISAK-K shows the following consistencies:

The factors of the ISAK-K “Time pressure”, “Problems in workflow due to supervisors and physicians”, “Problems in workflow due to other professional groups”, “Social support from direct supervisors”, “Social support from medical colleagues”, “Autonomy”, “Participation”, “Possibilities for further education” and “Possibilities for skill development” correspond well with our factors ***Quantitative Work Demands***, ***Social Relationships, Job Control, Opportunities for Participation and Professional Development*** and ***Variability***, respectively.

Concerning the remaining factors, consistencies are less obvious. The ISAK-K factor “Uncertainty” contains items like "How often does it happen that you have to take a decision, where consequences are difficult to predict?” One might interpret this as complexity, which is included in the KFZA factor ***Qualitative Work Demands*** in terms of “In my work, there are issues that are too complicated”. The ISAK-K factor “Frustration about how work needs to be done” contains items that describe a lack of time for patient care and for conversations with patients and relatives. The time aspect of this content is described by ***Quantitative Work Demands***, the emotional aspect by ***Emotional Demands.*** Items of the ISAK-K factor “Social stressors with patients” such as “How often does it happen that patients or their families reproach you?" correspond most likely to the items of the KFZA factor ***Emotional Demands***. However, factors consistent to the ISAK-K factors “Emotional dissonance” and “Justice” are not to be found in the KFZA, while in the ISAK-K there are no factors consistent to the KFZA factors ***Workplace Environment*** and ***Consequences of Strain***.

Comparison of our results with psychosocial factors that are listed in the guideline of the GDA [3] shows that except of the GDA factors “Responsibility”, “Work time” and “Physical factors” all other psychosocial factors are covered by the extended version of the KFZA. Especially the GDA factors “Emotional demands” and “Work equipment”, which were not covered by the original KFZA, are captured by the additional items of the factors ***Emotional Demands*** and ***Workplace Environment***. For further development of the KFZA, it might be important to note that items of ***Completeness of Task*** were difficult to assign to factors and might not be appropriate for the use in hospitals. While the GDA recommends that this psychosocial factor should be analyzed, it might be necessary to design more specific questions for the hospital setting. Subsequently, these items should be evaluated e.g., using cognitive interviewing. Concerning the items of *Work Disruptions,* item AU1 might be omitted as it is similar to item AM which describes the GDA factor “Work equipment” more specifically. In addition, the newly determined KFZA-factor ***Quantitative Work Demands*** corresponds well with the GDA factor “Work process” that includes possible critical attributes such as time pressure, work intensity and frequent interruptions. The items of the factor ***Consequences of Strain***, which are not psychosocial factors but a result of distress, are relevant due to a currently developed GDA checklist that is used by the supervisory authorities. Regarding to this checklist a company receives an additional credit point in the audit by surveying information about consequences of strain such as physical impairments.

### Limitations

Some limitations of this study should be noted. First, we had to exclude 568 questionnaires listwise (636 for the extended version) due to missing data as a result of varying questionnaire versions used. A comparison showed that the analyzed sample had a slightly but significantly different composition than the deleted one. Therefore, it cannot be ruled out that a factor analysis performed with the original sample would have yielded slightly different results. Another limitation might be that the content of the questionnaire is somewhat heterogeneous mixing psychosocial factors that may cause psychological distress and items representing consequences of strain. However, the latter items load on a distinct factor. Furthermore, the fact that sometimes items were left out due to the request by the work council might have led to selection bias, but we do not believe that any systematic effects were present in determining such decisions. In addition, the sample consisted of two occupational groups – physicians and nurses – whose specific working conditions have a lot in common but also show differences. The fact that the group of nurses outweighs the group of physicians may lead to bias, particularly as the ratio of nurses to physicians in our sample was higher than the national average. The factorial structure of our sample should be cross-validated in other samples of hospital workers. Furthermore, we only analyzed the aspect of the factorial validity. To complete the psychometric evaluation, further studies are necessary.

### Practical implications

The KFZA and its extended version are currently implemented to conduct Psychosocial Workplace Risk Assessments in hospitals. The results of such surveys are generally presented through the means of the original KFZA factors. Our study shows that this may not be entirely adequate, since it yielded specific factors for the use in hospital settings. Therefore, we suggest that the factors found should now be used as the basis for future analyses. Furthermore, items which have been shown as not appropriate for the use in hospitals should be rephrased and additional items might be generated to cover the missing GDA factors “Responsibility”, “Work time” and “Physical factors”. Further psychometric evaluation including cross-validation of the factorial structure in other samples should be performed to achieve an instrument for the use in hospital workplaces.

## Conclusion

Overall, our study shows the suitability of the KFZA, both in its original and in its extended version, for the use in hospital workers. However, instead of the 11 factors originally proposed by the authors of the KFZA on the basis of studies with office workers, a seven factor-solution appears to be a better fit when employed in hospitals. Even with the reduced number of factors we were able to replicate the overall content-related structure quite well. Some of the additional items of the extended KFZA version produced two additional factors whereas others were assigned to the existing factors. The extended version of the KFZA covers the psychosocial factors that are examined by the supervisory authorities to a greater extent but might be completed by further items. Our study contributes to the assessment of the validity of this popular instrument. It should be complemented by psychometric evaluation in further studies.

## Additional files

Additional file 1: Table S1. Work aspects, KFZA factors and items including additional items. (DOC 84 kb)

Additional file 2: Table S2. Descriptive statistics and internal consistencies of the scales of the extended KFZA. (DOC 36 kb)

Additional file 3: Table S3. Factors and items of the extended KFZA. (DOC 51 kb)

## Abbreviations

BAuA: Federal Institute of Work Safety and Occupational Medicine (Bundesanstalt für Arbeitsschutz und Arbeitsmedizin)
GDA: Joint German Occupational Safety and Health Strategy (Gemeinsame Deutsche Arbeitsschutzstrategie)
ISAK-K: Instrument for stress-related job analysis for hospital physicians (Instrument zur stressbezogenen Arbeitsanalyse für Klinikärztinnen und –ärzte)
JC: Job Control
KFZA: Short Questionnaire for Workplace Analysis (Kurzfragebogen zur Arbeitsanalyse)
OPPD: Opportunities for Participation and Professional Development
QLWD: Qualitative Work Demands
QNWD: Quantitative Work Demands
SR: Social Relationships
VAR: Variability
WE: Workplace Environment
