# Supplementary material for: Short Questionnaire for Workplace Analysis (KFZA): factorial validation in physicians and nurses working in hospital settings
Source: J Occup Med Toxicol. 2017 May 12;12:11. doi: 10.1186/s12995-017-0157-6 (PMC5429530; doi:10.1186/s12995-017-0157-6)
Supplement: Supplementary file 1 — Work aspects, KFZA factors and items including additional items. (DOC 84 kb) [file 12995_2017_157_MOESM1_ESM.doc]

**Additional Table S1** Work aspects, KFZA factors and items including additional items

| **Work aspects** | **KFZA factor** | **Items (item code)** |
| --- | --- | --- |
| Job Content | Variability | Learning new skills (VS1) |
|  |  | Use of knowledge, skills and ability (VS2) |
|  |  | Variety of tasks (VS3) |
|  | Completeness of Task | Visibility of task accomplishment (GH1) |
|  |  | Completeness of product (GH2) |
| Resources | Job Control | Influence on sequence of activities (HS1) |
|  |  | Influence on work content (HS2) |
|  |  | Influence on work load and procedures (HS3) |
|  | Social Support | Social support by co-workers (SR1) |
|  |  | Social support by supervisors (SR2) |
|  |  | Social cohesion within the department (SR3) |
|  | Cooperation | Necessity of cooperation (ZU1) |
|  |  | Opportunity for social exchange with co-workers (ZU2) |
|  |  | Feedback from supervisors and co-workers (ZU3) |
| Stressors | Qualitative  Work Demands | Excessive complexity of tasks (QL1) |
|  |  | Excessive demands on concentration (QL2) |
|  | Quantitative Work Demands | I frequently work under time pressure (QN1) |
|  |  | I have too much work to do (QN2) |
|  | Work Disruptions | Lack of information, work materials or equipment (AU1) |
|  |  | Interruptions of workflow (AU2) |
|  | Workplace Environment | Unfavourable physicochemical conditions (UB1) |
|  |  | Insufficient work space and equipment (UB2) |
| Organizational Culture | Information and Participation | Information about organizational developments (IM1) |
|  |  | Consideration of employee input (IM2) |
|  | Benefits | Continuous education (BL1) |
|  |  | Opportunities for advancement (BL2) |
|  | **Additional domains** | **Additional items (item code)** |
|  | Cooperation | Good cooperation between professional groups (ZU4) |
|  | Work Equipment | Adequate Work equipment (AM) |
|  | Emotional Demands | Social stressors with patients (EB1) |
|  |  | Time for communication with patients (EB2) |
|  |  | Excessive emotional demands (EB3) |
|  | Consequences of Strain | Severe exhaustion (FB1) |
|  |  | Difficulties unwinding after work (FB2) |
|  |  | Neck- / back- / shoulder-pain (FB3) |
|  |  | Disordered sleep (FB4) |
|  |  | Headaches (FB5) |
|  |  | Stomach and intestinal problems (FB6) |

*Abbreviations: KFZA* Kurzfragebogen zur Arbeitsanalyse (Short Questionnaire for Workplace Analysis)
