# Supplementary material for: Short Questionnaire for Workplace Analysis (KFZA): factorial validation in physicians and nurses working in hospital settings
Source: J Occup Med Toxicol. 2017 May 12;12:11. doi: 10.1186/s12995-017-0157-6 (PMC5429530; doi:10.1186/s12995-017-0157-6)
Supplement: Supplementary file 2 — Descriptive statistics and internal consistencies of the scales of the extended KFZA. (DOC 36 kb) [file 12995_2017_157_MOESM2_ESM.doc]

Additional Table S2 Descriptive statistics and internal consistencies of the scales of the extended KFZA

| Scales | Eigenvalues | M | SD | Cronbach’s α | Number of items |
| --- | --- | --- | --- | --- | --- |
| Social Relationships | 7.83 | 3.50 | 0.43 | 0.81 | 7 |
| Consequences of Strain | 3.83 | 3.31 | 0.44 | 0.83 | 6 |
| Job Control | 2.02 | 3.28 | 0.11 | 0.87 | 3 |
| Opportunities for Participation  and Professional Development | 1.88 | 2.84 | 0.57 | 0.76 | 4 |
| Quantitative Work Demands | 1.82 | 2.34 | 0.19 | 0.76 | 3 |
| Workplace Environment | 1.27 | 3.02 | 0.31 | 0.67 | 4 |
| Emotional Demands | 1.18 | 2.79 | 0.27 | 0.60 | 4 |
| Variability | 1.10 | 3.63 | 0.22 | 0.68 | 4 |
| Qualitative Work Demands | 1.06 | 3.62 | 0.17 | 0.63 | 2 |
