# Supplementary material for: Short Questionnaire for Workplace Analysis (KFZA): factorial validation in physicians and nurses working in hospital settings
Source: J Occup Med Toxicol. 2017 May 12;12:11. doi: 10.1186/s12995-017-0157-6 (PMC5429530; doi:10.1186/s12995-017-0157-6)
Supplement: Supplementary file 3 — Factors and items of the extended KFZA. (DOC 51 kb) [file 12995_2017_157_MOESM3_ESM.doc]

Additional Table S3 Factors and items of the extended KFZA

| **Factors determined**  **in this study** | **Items (item code)** |
| --- | --- |
| I Social Relationships | Social support by co-workers (SR1) |
|  | Social support by supervisors (SR2) |
|  | Social cohesion within the department (SR3) |
|  | Necessity of cooperation (ZU1) |
|  | Opportunity for social exchange with co-workers (ZU2) |
|  | Feedback from supervisors and co-workers (ZU3) |
|  | Good cooperation between professional groups (ZU4) |
| II Consequences of Strain | Severe exhaustion (FB1) |
|  | Difficulties unwinding after work (FB2) |
|  | Neck- / back- / shoulder-pain (FB3) |
|  | Disordered sleep (FB4) |
|  | Headaches (FB5) |
|  | Stomach and intestinal problems (FB6) |
| III Job Control | Influence on sequence of activities (HS1) |
|  | Influence on work content (HS2) |
|  | Influence on work load and procedures (HS3) |
| IV Opportunities for Participation and Professional Development | Information about organizational developments (IM1) |
|  | Consideration of employee input (IM2) |
|  | Continuous education (BL1) |
|  | Opportunities for advancement (BL2) |
| V Quantitative Work Demands | Workload (QN2) |
|  | Time pressure (QN1) |
|  | Interruptions of workflow (AU2) |
| VI Workplace Environment | Lack of information, work materials or equipment (AU1) |
|  | Unfavourable physicochemical conditions (UB1) |
|  | Insufficient work space and equipment (UB2) |
|  | Adequate Work equipment (AM) |
| VII Emotional Demands | Social stressors with patients (EB1) |
|  | Time for communication with patients (EB2) |
|  | Excessive emotional demands (EB3) |
|  | Completeness of product (GH2) |
| VIII Variability | Learning new skills (VS1) |
|  | Use of knowledge, skills and ability (VS2) |
|  | Variety of tasks (VS3) |
|  | Visibility of task accomplishment (GH1) |
| IX Qualitative Work Demands | Excessive complexity of tasks (QL1) |
|  | Excessive demands on concentration (QL2) |
